# Characterization of a Novel Peptide from Pathogenic *Leptospira* and Its Cytotoxic Effect

**DOI:** 10.3390/pathogens9110906

**Published:** 2020-10-30

**Authors:** Saksakon Paratsaphan, Saengduen Moonsom, Onrapak Reamtong, Sittiruk Roytrakul, Vanaporn Wuthiekanun, Nicholas P. J. Day, Piengchan Sonthayanon

**Affiliations:** 1 Department of Molecular Tropical Medicine and Genetics, Faculty of Tropical Medicine, Mahidol University, Bangkok 10400, Thailand; psaksakon@gmail.com (S.P.); onrapak.rea@mahidol.ac.th (O.R.); 2 Department of Protozoology, Faculty of Tropical Medicine, Mahidol University, Bangkok 10400, Thailand; saengduen.moo@mahidol.ac.th; 3 Proteomics Research Laboratory, National Center for Genetic Engineering and Biotechnology, Pathumthani 12120, Thailand; sittiruk@biotec.or.th; 4 Mahidol Oxford Tropical Medicine Research Unit, Faculty of Tropical Medicine, Mahidol University, Bangkok 10400, Thailand; lek@tropmedres.ac (V.W.); Nickd@tropmedres.ac (N.P.J.D.); 5 Centre for Tropical Medicine & Global Health, Nuffield Department of Clinical Medicine, University of Oxford, Oxford OX3 7FZ, UK

**Keywords:** novel peptide, pathogenic *Leptospira*, cytotoxicity

## Abstract

Leptospirosis is a zoonotic infectious disease caused by pathogenic *Leptospira* species. Virulence proteins have been shown to be key determinants of the pathogenesis of pathogenic *Leptospira*. A specific peptide at a mass-to-charge ratio of 7000 Da was identified in *Leptospira* whole cells using matrix-assisted laser/desorption ionization time-of-flight (MALDI-TOF) mass spectrometry. This peptide was specifically present in pathogenic *Leptospira* and in clinical isolates. We report here the characterization of this specific peptide using a proteomics approach. This peptide was significantly matched to a hypothetical conserved *L. interrogans* protein (LA2458) with a calculated molecular weight of 7140.136 Da containing a tellurite-resistance domain at its C terminus (TerB-C). The amino acid sequences revealed the presence of hydrophobic transmembrane portions and two linear B-cell epitopes. Despite its low abundance, this synthetic peptide demonstrated dose-dependent cytotoxicity toward African green monkey kidney (Vero) cells via the apoptosis pathway. The concentration of the peptide 100 µM induced about 50% of cell death after a 24 h exposure. This peptide could be useful for the diagnosis of leptospirosis and the study of pathogenesis.

## 1. Introduction

Pathogenic *Leptospira* species are the causative agent of leptospirosis, which is a widespread zoonosis with one million estimated cases that results in 60,000 deaths annually [1]. The number of leptospirosis cases is increasingly resulting from climatic events that result in heavy rainfall and flooding [2]. Humans are the accidental hosts of pathogenic *Leptospira* spp. Leptospires infection occurs through abraded skin. The invaded leptospires circulate via the bloodstream to multiple organs and may cause kidney damage and renal failure [3]. The symptoms of human leptospirosis may vary from mild flu-like symptoms to severe multi-organ failure. However, the mechanisms underlying the multi-organ failure caused by pathogenic *Leptospira* remain unclear. Proper therapeutics and vaccines may be the approach for controlling the leptospirosis burden. Virulent proteins associated with host cell damage and pathogenesis are the major focus of therapeutic and vaccine research [4]. The discovery of a factor associated with host-cell toxicity may provide insights into the mechanisms of pathogenesis of leptospirosis. The mechanisms by which damage occurs are not conclusively known [5]. There are studies that showed leptospiral membrane protein extracts induced an inflammatory response in cultured murine proximal tubule cells [6,7]. Recently, matrix-assisted laser/desorption ionization time-of-flight mass spectrometry (MALDI-TOF MS) was used to identify and differentiate *Leptospira* spp. The presence of eight peptide masses at 3502.33, 5776.50, 6352.06, 7006.38, 7969.51, 8734.66, 10,422.52, and 11,039.39 Da was unique to pathogenic *Leptospira*. In particular, a peptide found at a mass-to-charge ratio of 7006.38 Da exhibited a significantly high relative intensity in eight pathogenic *Leptospira* spp. and was detected in 97 *Leptospira* clinical isolates from Thailand and Laos [8]. In addition, this peptide was absent in intermediate and non-pathogenic *Leptospira* spp. Hence, it may represent a suitable biomarker of pathogenic *Leptospira* and be involved in leptospiral pathogenicity. This study aimed to identify and characterize the properties and roles of this peptide using a proteomics approach.

## 2. Results

### 2.1. Identification and Characterization of the Specific Peptide

**Figure 1:**
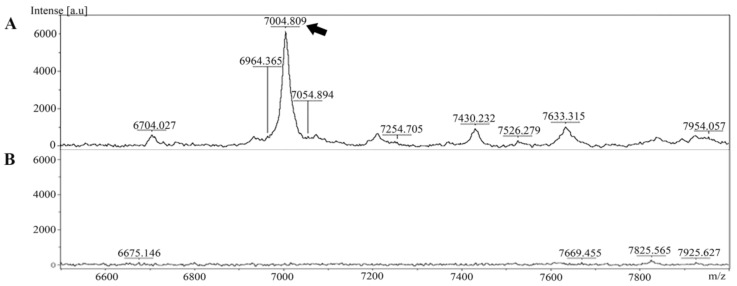
Mass spectra of the peptide extracted from *Leptospira* were generated using MALDI-TOF MS. (**A**) The peptide at approximately 7000 Da was present in the *Leptospira* cell pellet (indicated by the arrow). (**B**) Mass spectrum of the culture supernatant.

The extracted peptide from *Leptospira* whole cells was analyzed and identified in cell pellet and culture supernatant using MALDI-TOF MS. The specific peptide was found in the cell pellet but was absent in the culture supernatant (Figure 1).

**Figure 2:**
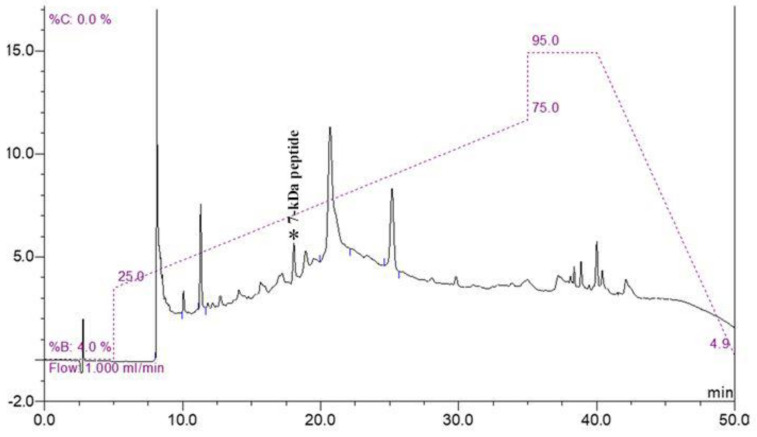
RP-HPLC chromatogram of purified peptide. Fraction containing 7 kDa peptide was eluted at a peak retention time of 18 min (peak marked by asterisk) on chromatogram.

Because of the complexity of other proteins in the cell pellet, the low-abundance peptide at 7000 Da was purified using molecular weight cut-off fractionation and RP-HPLC. All fractions were analyzed using MALDI-TOF MS. The fraction containing a 7 kDa peptide was eluted at a peak retention time of 18 min (Figure 2). The peptide was named Lp7, as it was isolated from *L. interrogans* with a size of approximately 7 kDa.

**Figure 3:**
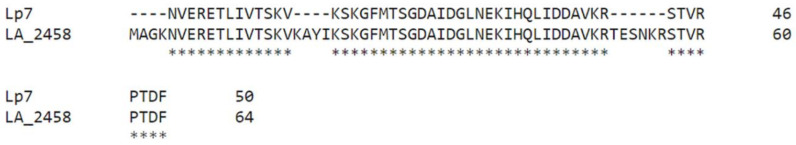
Comparison of Lp7 amino acid sequence to a hypothetical protein LA2458 of *L. interrogans* serovar Lai. An asterisk (*) indicates a position which has a single, fully conserved residue.

After purification, the peptide was identified using LC-MS/MS and MASCOT searching. The peptide was significantly matched (protein score, 376; *p*-value < 0.05) with a hypothetical conserved protein (LA2458) containing a tellurite-resistance domain at its C terminus (TerB-C), which is encoded by the *la2458* gene of *L. interrogans* serovar Lai strain 56601 (NCBI accession: AAN49657.2) [9], with 78% sequence coverage (Figure 3).

The calculated molecular weight of this peptide is 7140.136 Da. It comprises 64 amino acids with a theoretical isoelectric point (pI) of 9.45.

**Figure 4:**
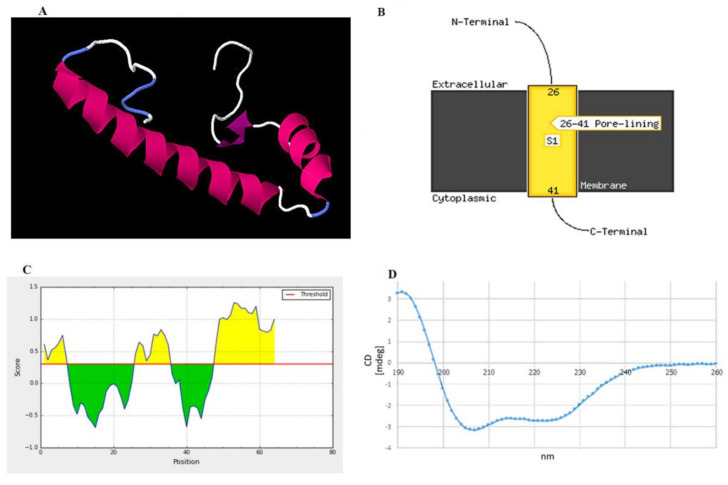
Structural prediction of the peptide identified in this study. (**A**) Proposed 3D structure in the built structure. Pink, purple, white, and blue represent helices, β-strands, and random coils, respectively. (**B**) Schematic transmembrane topology, as predicted based on amino acid sequences using the PSIPRED server, illustrating residues 26–41 as an α-helical transmembrane region and residues 1–25 (N terminus) and 42–64 (C terminus) as the extracellular and intracellular parts, respectively. S1 indicates the number of transmembrane regions. In this case, one transmembrane region was predicted. (**C**) B-cell epitopes predicted by the BepiPred server showing two epitopes at residues 1–7 and 25–36 as high probability to be linear B-cell epitopes. (**D**) Circular dichroism (CD) spectrum demonstrating the overall secondary structure of the synthetic peptide.

The three-dimensional structure of peptide was built and aligned with the Protein Data Bank (PDB) using a homology modeling approach. The crystal structure of the putative protein ttk003001566 (PDB ID: 1wwi) from *Thermus thermophilus* HB8 was used as a template based on the best *p*-value of the model (*p*-value = 3.05 × 10^−2^). The predicted secondary structures of the peptide were 51.56% α-helices, 12.50% β-strands, and 35.94% random coils, as shown in Figure 4A. The prediction using the PSIPRED tool demonstrated that the peptide had a transmembrane topology consisting of an extracellular region (residues 1–25), a membrane-lining helix (residues 26–41), and a cytoplasmic exposed region (residues 42–64) (Figure 4B). Furthermore, this peptide has two potential B-cell epitopes at amino acid residues 1–7 (MAGKNVE) and 25–36 (GFMTSGAIDGL) (Figure 4C). Using circular dichroism spectroscopy to demonstrate the secondary structure of this synthetic peptide, it revealed absorption patterns with maximal negative peaks at 207 and 223 nm, corresponding to an α-helix in the overall structure of the synthetic peptide in sodium phosphate buffer (50 mM, pH 6) shown in Figure 4D. The obtained circular dichroism (CD) spectra were further analyzed by the K2D3 and revealed 49.66% of α-helices and 3.74% of β-strands in their secondary structures.

### 2.2. Effect of the Synthetic Peptide on the Viability of Vero Cells

**Figure 5:**
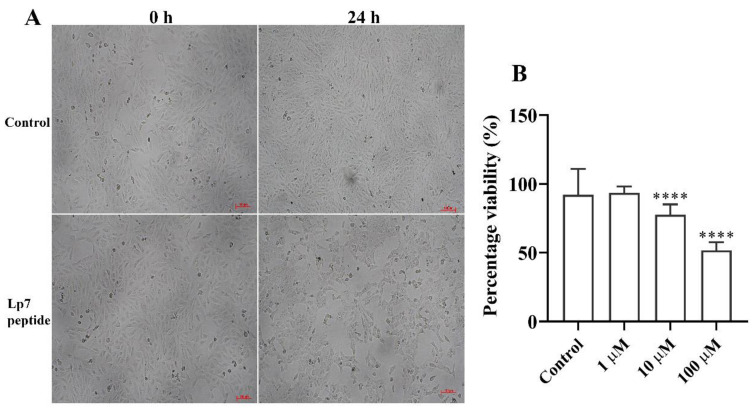
Gross morphology and viability of peptide-treated Vero cells. (**A**) Vero cells incubated for 24 h with 100 µM peptide. Gross morphology and cell confluence were imaged under an inverted microscope and compared with the controls. (**B**) Viability of Vero cells treated with the peptide for 24 h at concentrations of 1, 10, and 100 µM. Data are presented as the median and interquartile range (IQR) of six replicates from two independent experiments. **** *p*-value < 0.001, Kruskal–Wallis ANOVA. Scale bar, 100 µm.

The cytotoxicity of the peptide toward Vero cells was determined by observing changes in cell morphology and confluency using an inverted microscope and measuring cell viability via an MTT-based assay. Untreated Vero cells exhibited a fibroblast-like morphology (Figure 5A). After treatment with the synthetic peptide at concentrations of 1, 10, and 100 µM for 24 h, the Vero cells became round and clumped compared with cells before treatment and control cells. The cytotoxicity of the peptide in Vero cells was assessed based on 100% confluence of cells at 24 h after treatment to obtain reliable results. At this time point, the confluence of Vero cells exposed to 100 µM of peptide was decreased by about 50% compared with cells in the control group (Figure 5B). Cells showed 93.5%, 77.6%, and 51.7% viability after treatment with 1 µM, 10 µM, and 100 µM of peptide, respectively. At 48 h and longer, the confluences of the untreated cells were about 200% or higher, while the peptide treated cells could not reliably be analyzed. Thus, the 7-kDa peptide had significant (*p* < 0.001) dose-dependent cytotoxic activity toward Vero cells at 24 h post-exposure.

### 2.3. Induction of Apoptotic Programmed Cell Death by the Synthetic Peptide in Vero Cells

**Figure 6:**
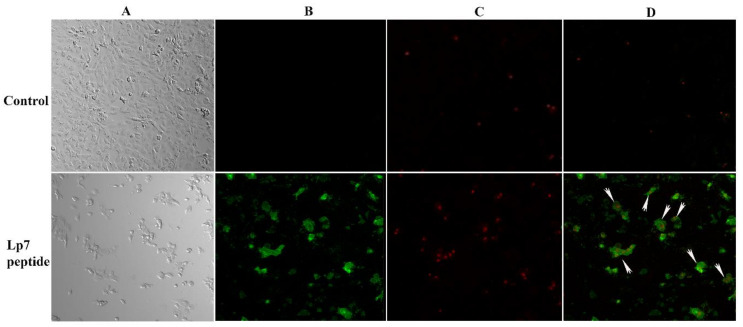
Confocal imaging of Vero cells treated with 100 µM synthetic peptide. Cells were treated with 100 µM peptide or control for 24 h. Cells were then double stained with annexin V-FITC and propidium iodide (PI). (**A**) Cells were visualized using a phase-contrast mode. (**B**) The sample was excited at 488 nm to visualize green fluorescence in cells stained with annexin V-FITC. (**C**) The sample was excited at 555 nm to visualize red fluorescence in cells stained with PI. (**D**) The double-stained cells (merged of annexin V-FITC and PI) are indicated by arrows.

To evaluate the mechanism of cytotoxicity of the peptide in Vero cells based on the phosphatidylserine apoptotic marker, annexin V and propidium iodide (PI) staining were applied to detect apoptotic and necrotic cells, respectively. After treatment with 100 µM synthetic peptide for 24 h, Vero cells were positive for both annexin V-FITC and PI, indicating that treated Vero cells were dying from late apoptosis (Figure 6).

### 2.4. Proteomics Profile of Vero Cells Treated With the Synthetic Peptide

**Figure 7:**
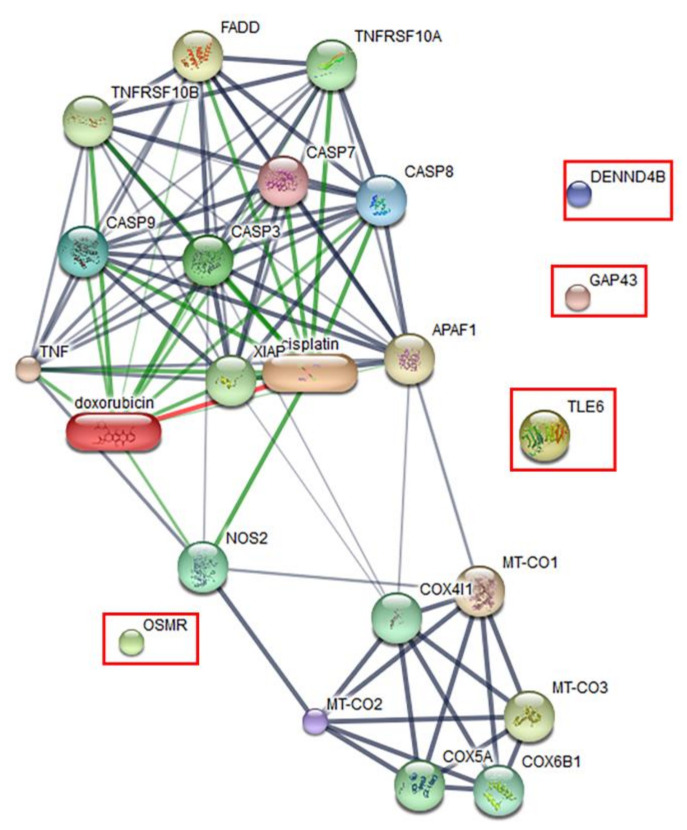
Predicted protein–ligand interaction pathways. The identified proteins (in the rectangles) were further analyzed using the STITCH server for pathways involved in peptide-mediated cytotoxicity in Vero cells.

The effect of the peptide on the proteomics profile change of Vero cells was investigated by mass spectrometry. A total of 1629 proteins were identified in the peptide-treated Vero cells. A total of 1515 proteins were commonly found in all treatment conditions. Forty-four proteins were found in both peptide-treated Vero cells and the positive control, while 66 proteins were present in both peptide-treated Vero cells and the negative control. Four proteins, including the DENN domain-containing protein 4B (DENND4B), neuromodulin (GAP43), oncostatin-M-specific receptor subunit beta (OSMR), and transducin-like enhancer protein (TLE6), were specifically expressed in peptide-treated Vero cells. These specific proteins were further defined by molecular functions and protein–protein network on apoptosis pathways. However, the STITCH protein–protein interaction demonstrated that they had no association with caspase pathways, which are the major cause of apoptotic cell death (Figure 7).

## 3. Discussion

The specific peptide generated by MALDI-TOF MS, which was found exclusively in pathogenic *Leptospira*, was successfully purified and identified using the proteomics approach. The peptide was presented in only cell pellet. This finding was also observed in another proteomics study [10]. Azad et al. demonstrated that there were 1073 proteins in a whole cell preparation and 540 proteins in a culture supernatant preparation of *L. interrogans* serovar Manilae strain L495 identified using Q Exactive Orbitrap mass spectrometry. Exoproteins involved in nutrient acquisition and metabolism were overrepresented, while other proteins in whole cell extracts have not been elucidated. The peptide in our study was presented in a cell pellet, which might suggest that the peptide could be a cell membrane-associated peptide. The peptide was small in size (64 amino acids) and with low abundance compared with total protein extracted from *L. interrogans* whole cells (data not shown). The amino acid sequences of this specific peptide retrieved by LC–MS/MS revealed their high similarity to a hypothetical protein of *L. interrogans* serovar Lai strain 56601. An in-silico protein analysis indicated that this peptide might serve as a novel peptide of pathogenic *Leptospira*. It also demonstrated a hydrophobic region anchored in the cell membrane with a surface exposure of the N terminus. Most of the virulence factors of pathogenic *Leptospira* are outer-membrane proteins such as LipL46 [11,12], Loa22 [13], and LipL21 [14,15]. However, the cellular localization of the peptide in *Leptospira* warrants additional laboratory investigation. The amino acid sequences of the peptide showed homology to the tellurite-resistance domain (TerB) of *L. interrogans*. Tellurite is a water-soluble form of tellurium that is highly toxic to bacterial species [16]. The tellurite-resistance determinant is important for the detoxification of tellurite and resistance to oxidative stress in many bacteria [17]. This determinant is present in a wide range of pathogenic bacteria and is frequently linked with other phenotypes, including resistance to bacteriophages and colicins [18]. However, there is little information regarding the role of this determinant in *Leptospira*. The peptide Lp7 also demonstrated a hydrophobic region anchored in the cell membrane with surface exposure of the N terminus. Structural analysis revealed a strong alpha-helical character of the novel peptide. Although the percentages of this helical component and beta strand obtained from CD spectra analysis were slightly different from those predicted from the 3D structure, it might be due to structural differences in the biologically mimicked and theoretical environments of the peptide in the CD analysis and 3D structure building, respectively.

Host-cell cytotoxicity is an important property of the virulence factors from pathogenic *Leptospira*, to cause leptospirosis. The cytotoxic processes occur after pathogenic *Leptospira* attach to host cells. It was shown that pathogenic *Leptospira* are capable of invading and inducing cytotoxicity in macrophage-like cells, including Vero or lung epithelial cells [19,20]. Furthermore, the hemolysin protein *of L. interrogans* exhibited comparable hemolytic and cytotoxicity toward epithelial cell lines derived from monkey (Vero) and human organelles and sheep erythrocytes [21,22]. Our study demonstrated that the Vero cell line is a choice for functional studies or screening of a novel protein or pathogen when pathogenesis is not completely known. The peptide used in this study was synthesized with low purity. However, there are several studies using peptides with 10% impurity or higher to assess the functional assay, and results could provide appropriate information of the cytotoxicity [23,24]. In addition, novel peptide of pathogenic *L. interrogans* exhibited a cytotoxic effect against a monkey kidney Vero cell line in a dose-dependent manner.

Apoptosis is programmed cell death that takes place to maintain cell homeostasis [25,26]. Apoptotic cells are determined by the staining of an apoptotic marker on a cell membrane using annexin V. Another characteristic of apoptotic cells is a rounded morphology, while cell membrane integrity remains intact [27]. Previous studies have demonstrated that the ability of pathogenic *Leptospira* to induce apoptosis in host cells could be determined by observing the morphological changes of *L. interrogans*-infected kidney cells [19,28]. The apoptosis inductivity of pathogenic *Leptospira* has been found to be associated with virulence proteins. Recently, several studies reported the role of the Fas-binding outer membrane protein and recombinant hemolysin (Sph2) of *L. interrogans* in the induction of apoptosis in macrophage, umbilical vein endothelium, embryo liver, and human lung epithelial cells [29,30,31]. Here, we demonstrated that Vero cells exposed to 100 µM peptide for 24 h developed late apoptosis. A comparative analysis of the protein–ligand interaction network demonstrated that four proteins were differently expressed in apoptotic Vero cells, but showed no association with the caspase pathway of apoptosis. It is possible that the late apoptosis induced in Vero cells by this peptide occurs through caspase-independent pathways. The four proteins expressed in Vero cells treated with peptide that were important to host cell response included DENND4B, which is a family of guanine nucleotide exchange factors (GEFs) and coordinates intracellular membrane trafficking events [32]; neuromodulin (GAP43), which is a nervous tissue-specific cytoplasmic protein and plays a key role in neuronal process, regeneration, and plasticity [33]; transducin-like enhancer protein 6 (TLE6), which is required for pre-implantation development [34]; and oncostatin M receptor (OSMR), which is a member of the type I cytokine receptor [35]. Furthermore, in-silico analysis of amino acid sequences revealed that this peptide harbors two B-cell epitope immunologic/antigenic sequences, suggesting that it might also play a role in the induction of the host antibody response.

In summary, a specific peptide Lp7 was identified from *L. interrogans* cell extracts as a 64-mer predicted to have a strong alpha helical character. The synthetic peptide Lp7 demonstrated cytotoxicity toward Vero cells; it was further characterized using a proteomic approach to reveal host response to a peptide. The current results indicate that this novel peptide of pathogenic *Leptospira* plays roles in inducing apoptotic programmed cell death.

## 4. Materials and Methods

### 4.1. Leptospira Cultivation

The *Leptospira interrogans* serogroup Icterohaemorrhagiae serovar Lai strain LR31 used in this study was obtained from the WHO/FAO/OIE Collaborating Centre for Reference and Research on Leptospirosis, Queensland, Australia. The isolate was grown under aerobic conditions in liquid medium (Ellinghausen, McCullough, Johnson, and Harris (EMJH) broth; Difco, Becton Dickinson, USA) supplemented with 3% normal rabbit serum at 30 °C for 7 days.

### 4.2. Extraction and Purification of the Peptide

Cultured *Leptospira interrogans* cells were pelleted by centrifugation at 10,000× *g* at 4 °C for 15 min. The culture supernatant was kept for the analysis of secreted proteins. The cell pellet was washed with 70% ethanol (Merck Millipore, Burlington, MA, USA). Proteins were extracted with a solution containing 2.5% trifluoroacetic acid (TFA) and 50% acetonitrile (Sigma-Aldrich, St Louis, MO, USA). The cell lysate was centrifuged at 13,000× *g* at 4 °C for 2 min, and the supernatant containing the crude protein extract was collected for further purification and analysis. The proteins in the culture supernatant were precipitated using pre-chilled acetone. After centrifugation at 10,000× *g* at 4 °C for 15 min, the supernatant was discarded, and the protein pellet was suspended in 2.5% TFA in 50% acetonitrile and subjected to MALDI-TOF mass spectrometry, to assess the secreted proteins. Protein concentration was determined in the cell pellet and secretory portion using Bradford’s assay (Bio-Rad Laboratories, Hercules, CA, USA). To analyze the presence of the 7-kDa peptide in the cell pellet and culture supernatant, MALDI-TOF mass spectrometry (AutoFlex Speed, Bruker Daltonics, Bremen, Germany) was performed as described previously [8]. Briefly, 1 µL of protein solution was spotted onto a MALDI target plate and mixed with 1 µL of sinapinic acid (SA) matrix solution (10 mg/mL SA in 0.1% TFA, 70% acetonitrile) (Bruker Daltonics). MALDI-TOF mass spectra were acquired in the linear positive mode using an AutoFlex Speed instrument (Bruker Daltonics) with FlexControl software (to control the instrument). The detecting spectra were set in the mass range of 2000–20,000 Da using 80% laser energy. The spectral representative of each spot was collected and analyzed from a total of 5000 laser shots using FlexAnalysis software (Bruker Daltonics).

The crude protein extract was fractionated through a 10 kDa molecular weight cut-off (MWCO) filter (Macrosep Advance, PALL, USA) to reduce protein complexity. The flow-through fraction containing the 7-kDa peptide peak was collected for further purification by reversed phase high-performance liquid chromatography (RP-HPLC). The flow-through fraction of 10-kDa MWCO was centrifuged at 3000× *g* for 1 h and injected into an HPLC system (Dionex Ultimate 3000 UHPLC, Thermo Scientific, Sunnyvale, CA, USA) equipped with a reversed phase C18 column (Acclaim 120 C18 column; particle size, 5 µm; pore diameter, 120 Å; 4.6 × 150 mm; Thermo Scientific) and maintained at 40 °C. The mobile phases were 0.1% (*v*/*v*) trifluoroacetic acid (TFA) in water (mobile phase A) and 0.1% (*v*/*v*) TFA in acetonitrile (mobile phase B). Proteins were eluted using a linear gradient of 25–75% mobile phase B at a flow rate of 1 mL/min for 30 min. Proteins in the eluted fractions were detected using an ultraviolet (UV) detector at a wavelength of 280 nm. All collected fractions were dried using a centrifugal evaporator (TOMY Centrifugal Concentrator CC-105, Tokyo, Japan) and analyzed for the presence of the 7-kDa peptide by MALDI-TOF mass spectrometry. A fraction containing the 7-kDa peptide target was further separated by 15% Tris-Tricine SDS–PAGE as described previously [36] using a mini Protean III gel apparatus (Bio-Rad Laboratories). After electrophoresis, the gel was fixed in 45% methanol, 10% acetic acid for 30 min, and stained with Coomassie Brilliant Blue G-250 for 1 h. The gel was then destained with 45% methanol, 10% acetic acid until the background was clear, and the band with a relative molecular mass (M_r_) of approximately 7000 Da was cut out and digested with trypsin, as described by Shevchenko [37]. In brief, proteins in the gel slices were reduced with 10 mM dithiothreitol (DTT), then alkylated with 250 mM iodoacetamide (IAM) (Sigma-Aldrich). Finally, the reduced and alkylated proteins were digested with sequencing-grade trypsin (Sigma-Aldrich) at 37 °C overnight for further identification.

### 4.3. Protein Identification by Liquid Chromatography–Tandem Mass Spectrometry (LC–MS/MS)

The in-gel tryptic digested peptides were injected into a nanoLC system (DIONEX, Ultimate 3000, UHPLC, RSLCnano, Thermo Scientific, Sunnyvale, CA, USA) and separated by a reversed-phase C18 PepMap column (75 µm × 15 cm; diameter, 2 µm; pore size, 120 Å) (Acclaim PepMap, Thermo Scientific, Sunnyvale, CA, USA). The nanoLC system was equipped with an electrospray quadrupole time-of-flight mass spectrometer (micrOTOF-Q II, Bruker Daltonics, Bremen, Germany). Peptides were eluted from the reversed-phase C18 column into the mass spectrometer using a linear gradient of 5–55% mobile phase B (0.1% formic acid in 80% acetonitrile) for 30 min at a flow rate of 0.3 µL/min. The data acquired by tandem mass spectrometry were processed using a data analysis software package (Bruker Daltonics GmbH). The processed data were used to identify proteins in a public database using the MASCOT server version 2.3 (Matrix Science, London, UK) by searching against the *L. interrogans* sequence database. The search parameters of MASCOT were set as follows: peptide mass tolerance, ±1.2 Da; MS/MS tolerance, ±0.6 Da; and variable modifications were set at carbamidomethylated cysteine and oxidized methionine residues.

### 4.4. Characterization of the Novel Peptide Sequence

The amino acid sequence of the novel peptide based on sequence alignment to the MASCOT database was synthesized with 90.78% purity (KareBay Biochem, Inc., South Brunswick Township, NJ, USA) [23,24]. The secondary structure of the synthetic peptide was analyzed by circular dichroism (CD) spectroscopy. In brief, 100 µg/mL of the synthetic peptide was dissolved in 50 mM sodium phosphate buffer (pH 6), added to a 1 mm path length quartz cuvette, and measured using a spectropolarimeter (Jasco J-815 spectrometer, Tokyo, Japan) with a scanning wavelength of 190–260 nm at 25 °C. All spectra represent the average of five scans. The obtained CD spectra were further analyzed by K2D3 tool [38] to estimate secondary structure of synthetic peptide. The three-dimensional (3D) structure was built from its amino acid sequences using the homology modeling of the RaptorX server (http://raptorx.uchicago.edu/StructurePrediction/predict/) [39,40]. The QMEAN score (http://swissmodel.expasy.org/qmean/cgi/index.cgi) [41,42] and the Ramachandran plot obtained using the RAMPAGE server (http://mordred.bioc.cam.ac.uk/~rapper/rampage.php) [43] were used to evaluate the accuracy of the model built here. The signal peptide in the peptide sequence was predicted using the SignalP server (http://www.cbs.dtu.dk/services/SignalP/) [44] and Phobius signal prediction [45,46]. Physicochemical properties, including molecular weight and theoretical pI, were calculated using the Compute pI/Mw tool (ExPASy) [47,48]. The PSIPRED server and the MEMSAT-SVM method were used to predict membrane helices for, and the topology of, the peptide. The linear B-cell epitopes were predicted by the BepiPred 2.0 tool (http://www.cbs.dtu.dk/services/BepiPred/), which relies on the Hidden Markov model [49].

### 4.5. Cytotoxicity Testing on Vero Cells

To measure the cytotoxic potential of the synthetic peptide toward monkey kidney epithelial cell line or Vero cells (ATCC CCL-81), 3-(4,5-dimethylthiazol-2-yl)-2,5-diphenyltetrazolium bromide (MTT) metabolic activity assay was applied as described previously [50]. Vero cells were plated in 96-well plates at 6000 cells/well in Eagle’s minimum essential medium (MEM) supplemented with 10% fetal bovine serum (HyClone, GE Healthcare Life Science, Logan, UT, USA) at 37 °C with 5% CO_2_ using six replicates per condition. After 24 h of culture, the medium was discarded and replaced with the synthetic peptide prepared in MEM at various concentrations (0, 1, 10, and 100 µM). After 24 h of incubation, 10 µL of MTT solution (5 mg/mL in PBS, AppliChem GmbH, Darmstadt, Germany) was added and incubated for an additional 4 h at 37 °C with 5% CO_2_. After medium was removed, the mixture of 4 mM HCl and 0.1% Nonidet P-40 in isopropanol was added to dissolve formazan crystals. The optical density (OD) of the dissolved formazan was measured at 590 nm, with a reference wavelength of 620 nm. The percentage of cell viability was calculated by comparing the OD of the treated groups with that of the untreated control. Descriptive statistics were used to describe the percentage viability of each treatment. Kruskal–Wallis analysis of variance (ANOVA) followed by Dunn’s multiple comparison were used to compare the differences between the treated and control groups. Significance was set at *p*-value < 0.05. Statistical analysis was performed using GraphPad Prism version 7.0 for Windows (GraphPad Software, San Diego, CA, USA).

To determine the effect of the peptide on programmed cell death, Vero cells were cultured with 100 µM of the synthetic peptide for 24 h, stained with fluorescein isothiocyanate (FITC)-conjugated annexin V, and counter stained with propidium iodide (Invitrogen, Eugene, OR, USA). Apoptotic and necrotic cells were visualized using confocal fluorescence microscopy (LSM 700; Carl Zeiss, Jena, Germany).

### 4.6. Comparative Proteomics Study of the 7-kDa-Peptide-Treated Vero Cells

Comparative proteomics was performed to analyze the proteome changes of Vero cells treated with the synthetic peptide. Vero cells were treated using three conditions: (1) 100 µM synthetic peptide; (2) 10% dimethyl sulfoxide (DMSO) (Merck, Burlington, MA, USA), as a positive control; and (3) 50 mM sodium phosphate buffer in MEM, as a negative control for 24 h. Treated cells were lysed with lysis buffer containing 0.5% SDS and reduced with 20 mM dithiothreitol (DTT). Subsequently, the protein lysate was alkylated with 100 mM iodoacetamide (IAM). The reduced and alkylated protein lysate was then digested with sequencing-grade modified trypsin (Promega, Madison, WI, USA) using a trypsin-to-protein ratio of 1:20 at 37 °C overnight. Trypsin-digested peptides were diluted with 0.1% (*v*/*v*) formic acid and injected into a reversed-phase column (PepSwift monolithic column, 100 μm i.d. × 50 mm) coupled to an UltiMate 3000 UHPLC system (Dionex, Thermo Scientific). Peptides were eluted to an ESI ion trap MS HCT ultra PTM Discovery system mass spectrometer (Bruker Daltonics). Mass spectra were compared among the different treatment conditions using the DeCyder MS 2.0 Differential Analysis software (GE Healthcare, Piscataway, NJ, USA). All MS/MS data were searched against the MASCOT server, version 2.0 (Matrix Science). The search parameters were as follows: fixed modification of carbamidomethyl at cysteine and oxidation at methionine residues; monoisotopic mass tolerance, ±0.5 Da; and 1 missed trypsin cleavage. The identified proteins were mapped regarding molecular functions and protein–protein network pathways using the STITCH server (http://stitch.embl.de/).
